# Effectiveness and response differences of a multidisciplinary workplace health promotion program for healthcare workers

**DOI:** 10.3389/fmed.2022.930165

**Published:** 2022-07-26

**Authors:** Kai-Hung Cheng, Ning-Kuang Wu, Chao-Tung Chen, Chih-Yu Hsu, Yen-An Lin, John Jiin-Chyuan Luo, Li-Ang Lee, Hai-Hua Chuang

**Affiliations:** ^1^Department of Family Medicine, Chang Gung Memorial Hospital, Taoyuan, Taiwan; ^2^Department of Education, Chang Gung Memorial Hospital, Taoyuan, Taiwan; ^3^Department of Family Medicine, Chang Gung Memorial Hospital, Kaoshiung, Taiwan; ^4^College of Medicine, Chang Gung University, Taoyuan, Taiwan; ^5^Department of Occupational Medicine, New Taipei Municipal Tucheng Hospital, Chang Gung Memorial Hospital, New Taipei City, Taiwan; ^6^School of Medicine, National Tsing Hua University, Hsinchu, Taiwan; ^7^Department of Otorhinolaryngology-Head and Neck Surgery, Chang Gung Memorial Hospital, Taoyuan, Taiwan; ^8^Health Promotion Center, Chang Gung Memorial Hospital, Taoyuan, Taiwan; ^9^Department of Industrial Engineering and Management, National Taipei University of Technology, Taipei, Taiwan

**Keywords:** anthropometrics, exercise intervention, hospital employee, multidiscipline, physical fitness, shiftwork, workplace health promotion

## Abstract

**Background:**

Workplace health promotion (WHP) in the healthcare industry is an important yet challenging issue to address, given the high workload, heterogeneity of work activities, and long work hours of healthcare workers (HCWs). This study aimed to investigate the effectiveness and response differences of a multidisciplinary WHP program conducted in HCWs.

**Methods:**

This retrospective cohort study included HCWs participating in a multidisciplinary WHP program in five healthcare facilities. The 20-week intervention included multiple easy-to-access 90-min exercise classes, one 15-min nutrition consultation, and behavioral education. Pre- and post-interventional anthropometrics, body composition, and physical fitness (PF) were compared with paired sample *t*-tests. Response differences across sex, age, weight status, and shiftwork status were analyzed with a generalized estimating equation.

**Results:**

A total of 302 HCWs were analyzed. The intervention effectively improved all anthropometric (body mass index, waist circumference, waist-hip ratio, and waist-to-height ratio), body composition (body fat percentage, muscle weight, visceral fat area), and PF (grip strength, high jump, sit-up, sit-and-reach, step test) parameters in all participants (all *p* < 0.05). Subgroup analyses revealed shift workers had a more significant mean reduction in body mass index than non-shift workers (adjusted *p* = 0.045). However, there was no significant response difference across sex, age, and weight subgroups.

**Conclusion:**

This study suggested that a multidisciplinary WHP program can improve anthropometric and PF profiles regardless of sex, age, and weight status for HCWs, and shifter workers might benefit more from the intervention.

## Introduction

While focusing on preserving people’s health as their work duties, healthcare workers (HCWs) themselves are also in need of health promotion (1, 2). The literature has indicated that unfavorable working conditions, such as night shifts, long work hours, psychosocial job strain, and job insecurity, can lead to negative health outcomes, including obesity (3), metabolic syndrome (4), muscular-skeletal discomforts (5), mental disorders (6, 7), and certain cancers (8–13). It is not uncommon that HCWs have to work under stressful conditions with long hours and irregular shifts, which may have negative impacts on their health (14, 15). HCWs have been reported to have higher levels of sickness absence, work dissatisfaction, distress and burnout than workers in other industries (16).

The importance of workplace health promotion (WHP) is well recognized (17). Previous studies have suggested that WHP can lead to lower disease prevalence, reduced medical costs, and higher productivity (18–22). However, the high workload, heterogeneity of work activities, and long work hours can make hospitals a difficult setting to address WHP (23). Moberg et al. found significant associations between physical activity, physical capacity (defined with maximal oxygen uptake (VO_2max_) and handgrip strength), and levels of musculoskeletal pain among HCWs (24). The same association was not observed in construction workers, suggesting that HCWs might be a population more likely to benefit from exercise-based WHPs (24).

Moreover, effectiveness of and predictors for the responses to WHP are not yet well understood (25–27). A systematic review conducted in 2012 showed positive effects of WHP on physical activity, diet, and body mass index (BMI) but insufficient evidence on absenteeism and mental health (28). According to a randomized clinical trial conducted by Reif et al., a significantly higher proportion of employees reported having a primary care physician and showed improvements in a set health belief in the treatment group of a 24-month WHP; however, no significant differences were observed in biometrics, medical diagnoses, or medical use (29). After an 18-month WHP in a clustered randomized trial, Song and Baicker found some positive self-reported health behaviors among the employees exposed but no significant improvement in health measures, healthcare spending and utilization, or employment outcomes (29). Another randomized controlled trial conducted by Patti and colleagues demonstrated that, subjects participating in different exercise programs differed in their improvements of physical performance (30). Mixed evidence indicates that WHP interventions can be somewhat effective (31), but it is still inconclusive and needs more research to confirm the benefit.

The global pandemic of COVID-19 has caused an unprecedented impact on healthcare systems worldwide, and research interests in the safety and health of HCWs have been growing ever since (32, 33); however, most focused on the infection of COVID-19 (34), vaccination against COVID-19 (35, 36), or mental health issues (37, 38). The latest review on HCWs health promotion was conducted by Brand et al., which included only 11 studies with system approaches (16). Data on effectiveness and response differences of WHP among HCWs remain insufficient. Validating WHP programs designed for use in hospitals and exploring the factors that influence intervention outcomes would benefit future design and implementation. This study examined the effectiveness of a multidisciplinary 20-week WHP program in northern Taiwan and analyzed response differences by sex, age, weight, and shiftwork statuses.

## Materials and methods

### Study design

This study was a retrospective case series to assess the effectiveness and response differences of a multi-site and multidisciplinary WHP program regarding anthropometrics, body composition (BC), and physical fitness (PF) of hospital employees. The original study was approved by the Institutional Review Board of the Chang Gung Memorial Foundation (No. 102-4004B). Informed consent was obtained from each subject. This study followed the guidelines of the World Medical Association Declaration of Helsinki (39) and the template for intervention description and replication (TIDieR) (40).

### Participants

Participants were recruited to the WHP program *via* posters, emails, and e-bulletin systems between February 23rd to March 22nd, 2015, from three hospitals and two nursing centers of Chang Gung Medical Foundation in northern Taiwan. The participants had to primarily meet the following criteria: (1) employees of the aforementioned five healthcare facilities of Chang Gung Medical Foundation, (2) aged between 18 and 64 years, and (3) voluntary participation in the program. Participants with the following condition were excluded: self-reported history of stroke, coronary artery disease, or other major vascular diseases.

Basic information, anthropometrics, BC, and PF were assessed before and after the single-arm intervention program. Participants who did not complete the intervention or had no available pre- and post-interventional data of BC and PF were not eligible for statistical analysis. Unless the above conditions, all cases that met the inclusion criteria would be included.

### Intervention – multidisciplinary program

**FIGURE 1:**
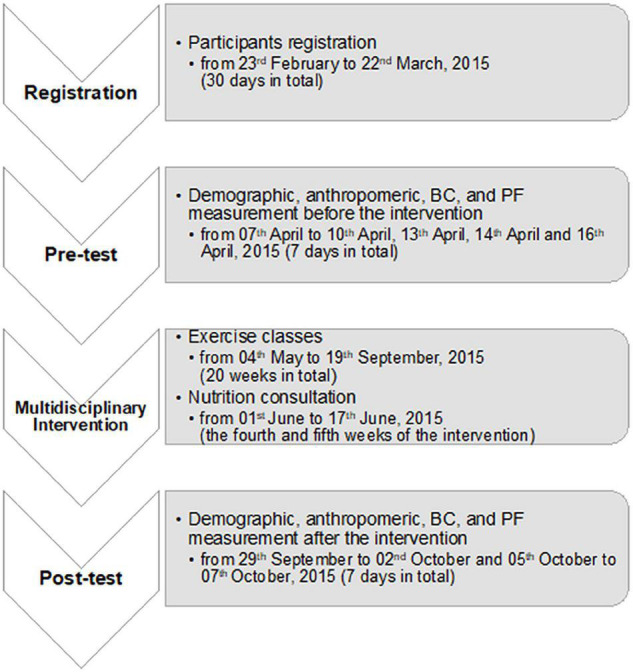
Flow chart of the multidisciplinary. BC, body composition; PF, physical fitness.

The program was conducted by a multidisciplinary team, including family physicians, rehabilitation physicians, sport science experts, PF trainers, clinical dietitians, and research assistants. A diagrammatic overview of the intervention is presented in Figure 1.

The intervention was workplace-based, 20 weeks long, and multi-faceted, comprising three parts:

#### Exercise classes

Up to 24 group exercise classes per week (including 5 in the morning and 19 in the evening) were held during the intervention period. Styles of the classes included aerobic dance, kickboxing, cycling, and Yogalates. Each class had a limit on the number of participants, depending on the characteristic of the exercise style and the size of the venue. The sizes of venues ranged from 73 to 165 square meters. The limit number of classes ranged from 15 to 40 participants. Each participant was requested and allowed to register for one class per week in a first-come, first-served manner. A class would be closed for registration once the limit number of participants was reached.

All sessions were held either in or nearby the study hospitals and instructed by certified trainers. Every exercise class was 80 min, including stretching and warming up for the first 20 min, main exercise for 50 min, and cool down for the last 10 min. The intensities of classes varied. All courses were gender-neutral. Best efforts were made to ensure the accessibility of the exercise program by offering multiple choices of time, location, style, and intensities of the classes so that participants could easily find ones fitted into their schedule and preference.

In aerobic dance classes, the PF trainer leads the participants to dance to lively music. In kickboxing classes, participants boxed under a PF trainer’s instruction, and the speed of boxing was emphasized to increase instantaneous velocity. Participants in cycling classes rode on spinning bikes under the guidance of the trainer. Yogalates was performed with participants doing yoga with a yogic instructor. The sustaining posture in yoga focused on the participants’ muscle endurance and respiration.

#### Nutrition consultation

A 15-min one-on-one nutrition consultation session by professional clinical dietitians was provided to participants with overweight or obesity, excessive body fat percentage (BF%), or metabolic syndrome. Overweight was defined as BMI > 24 according to the Ministry of Health and Welfare in Taiwan (41). Excessive body fat percentage was defined as BF% > 20% in males and BF% > 30% in females, according to the Sports Administration, Ministry of Education in Taiwan (42, 43). Metabolic syndrome was defined as the presence of three or more of the following five risk factors: (1) Abdominal obesity: (waist circumference: male ≥ 90 cm, female ≥ 80 cm). (2) Hypertension: systolic blood pressure (SBP) ≥ 130 mmHg/diastolic blood pressure (DBP) ≥ 85 mmHg. (3) Hyperglycemia: fasting blood glucose ≥ 100 mg/dl. (4) High-density lipoprotein cholesterol: male < 40 mg/dl, female < 50 mg/dl. (5) High triglyceride ≥ 150 mg/dl. The criteria were determined by the Ministry of Health and Welfare in Taiwan (41).

Nutrition consultation sessions were arranged in the fourth and fifth weeks of the intervention. The anthropometrics and BC of the individuals were provided to dietitians before the consultation by the assistants. During a consultation, the dietitian would ask the participant what he or she typically ate for breakfast, lunch, dinner, and late-night supper. After a dietitian learned about the participant’s nutritional status and daily lifestyle routine, the dietitian would make personalized suggestions according to each participant’s weight status, BC and personal concerns. For instance, instructions for participants with overweight or obesity would be focused on a healthy diet with a negative caloric balance. Participants with an unbalanced diet would learn about macronutrients. Individuals with low MW or high VFA would be given suggestions on the ratio of macronutrients and types of exercise.

#### Behavioral education

Multiple campaign activities were done to promote the WHP based on essential principles of the theory of planned behavior, namely behavioral beliefs, normative beliefs, and control beliefs (self-efficacy) (44). To increase *behavioral beliefs*, educational materials on exercise, PF, and weight management were delivered through emails, website bulletin boards, and posters in the workplace (Supplementary Figures 1–4). The educational materials were all designed by the multidisciplinary team. Messages encouraging HCWs to exercise were sent intensively by assistants and PF trainers during the intervention to enhance *normative beliefs*. For example, PF trainers were requested to publicize the benefits of exercise during the classes to increase their exposure to information on healthy behaviors. Moreover, by having a big ceremony to celebrate the success of the program and publicly reward participants with high attendance rates and considerable physical improvements, the intervention was also able to increase the *self-efficacy* of participants in terms of performance attainment (mastery experience), vicarious experience, and verbal persuasion.

### Measurements

#### General characteristics

Participants’ general characteristics were assessed by completing registration sheets at participants’ entry into the study. The following characteristics were assessed: age, sex, medical history, profession (nurse, medical technician, administration staff, and others), and shift status [day-shift only or night-shift only (i.e., non-shift workers); irregular shift work (shift workers)].

#### Anthropometrics

Body height, body weight, waist circumference (WC), and hip circumference (HC) were measured and used to calculate body mass index (BMI), waist-hip ratio (WHR), and waist-to-height ratio (WHtR).

#### Body composition

BC was measured using bioelectrical impedance analysis, which is based on differences in the conductivity of various components of the human body. We used the IOI-353 BC analyzer (Jawon Medical, Yuseong, South Korea) for the analysis, measuring segmental multi-frequency impedance values at 1, 5, 50, 250, 550, and 1,000 kHz with a tetra-polar 8-point tactile electrode system (45). Variables assessed included body fat percentage (BF%), muscle weight (MW), and visceral fat area (VFA).

#### Physical fitness

PF was tested as follows. The assessments of PF were implemented in accordance with the protocols developed and published by the Sports Department, Ministry of Education in Taiwan (46). All the assessments were completed by board-certificated exercise trainers.

Explosive muscle strength of the upper limbs was assessed with handgrip strength. Participants put their hands by the body side in a standing position. Then they were asked to grasp the handgrip by the dominant hand and gripped it instantaneously with their biggest strength. Two measurements were done to get the best record (47).

Explosive muscle strength of the lower limbs was assessed with vertical jumping. Vertical jumping was measured by participants jumping from the floor with both feet and touching the ruler with a hand as tall as they could. Two measurements were required to get the tallest value (48).

Muscular endurance was assessed with 1-min sit-up. Participants did 1-min sit-up with their upper body lying on the ground and knees had a 90-degree angle. Their feet were flat on the floor and PF trainers would lightly press on the feet to help stabilize. As a preparation position, participants had their forearms crossed to touch the contralateral shoulder and elbows upon the chest. Participants were asked to contract abdominal muscles to get up and touch the knees with elbows. After they returned to the preparation position, it was considered a count. PF trainers recorded the number of completions in 1 min (46, 49).

Flexibility was assessed with sit-and-reach technique. Participants sit on the ground with knees straight and toes faced upward. Their two heels were 30 cm apart and aligned with the 25 cm on the measured ruler. Participants leaned forward their upper body and stretched their hands to touch the ruler. After the folded two middle fingers touched the ruler, participants were asked to hold for 2 s to record. The measurement was conducted twice to get the best value (46, 50).

Cardiorespiratory fitness was assessed by 3-min step test, defined as the time that heart rate returns to normal after exercise. Each participant got on and off the bench for 3 min in continuous while maintaining a consistent pace. The height of the wooden bench was 35 cm, and the consistent pace was 96 beats per minute (46, 51).

### Statistical analysis

All study parameters were compared before and after the intervention using paired sample *t*-tests when dealing with the whole cohort. Using the Shapiro–Wilk test to assess the normality, descriptive statistics were expressed as mean [standard deviation (SD)] for normally distributed continuous variables, median [interquartile range (IQR)] for skewed variables, and number (proportion) for categorical variables. For within-group comparisons, the paired-samples *t*-test or Wilcoxon signed rank test was used as appropriate. For between-group comparisons, the independent samples *t*-test or Mann–Whitney *U* test was used as appropriate.

Generalized estimating equations (GEEs) were used to compare the differences in the changes of these parameters by cohort subgroups, including sex, age, weight status, and shift-work status. Specifically, each subgroup variable was entered into separate GEEs that included main effects of time (post-intervention vs. pre-intervention) and subgroup variable (e.g., male vs. female), a two-way interaction term of time × subgroup variable, and the main effects of baseline characteristics. A significant interaction effect signified a difference in change between subgroups.

All *p*-values were two-sided and statistical significance was accepted at *p* < 0.05. The data analyses were carried out using IBM SPSS Statistics 25 (International Business Machines Corp., Armonk, NY, United States) and Graph Pad Prism 9.0 for Windows (Graph Pad Software Inc., San Diego, CA, United States).

## Results

### Characteristics of study participants

**FIGURE 2:**
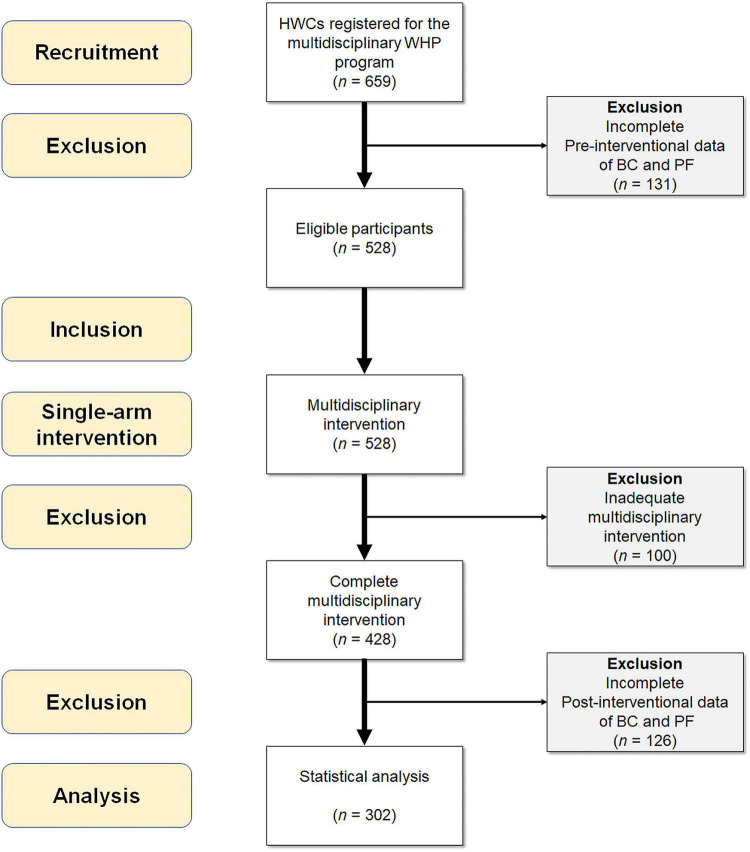
Inclusion flow chart of the study participants. BC, body composition; PF, physical fitness; WHP, workplace health promotion.

During the recruitment period, 659 HCWs registered for the WHP program, among whom 528 eligible participants completed pre-tests. Each participant was encouraged to attend one exercise class per week for 20 weeks (20 classes in total). To assure the internal validity of investigations on effectiveness and response differences of the intervention, those with a participation rate lower than 40% (8 classes) were excluded (*n* = 100). Participants who had no complete data of post-interventional assessments were further excluded (*n* = 126). A total of 302 subjects were enrolled for statistical analysis (Figure 2).

Among the 302 participants, median age was 36 years (IQR: 30–45) (Table 1), and most (93.0%) were female. Furthermore, 118 (39.1%) participants were older than 40 years of age. The median BMI was 22.6 kg/m^2^ (IQR: 20.9–24.7) and 107 (35.4%) of them were overweight (according to a BMI of greater than 24 kg/m^2^). Administrative staff and others (45.4%) and nurses (39%) were predominant among participants’ professions, and 47 (15.6%) participants served as medical technicians. Approximately one-third (32.8%) of the participants engaged in shiftwork.

**TABLE 1:** Characteristics of the study participants.

Variable	Overall	Male	Female	*p*-value^1^
*N*	302	21	281	
Age, years	36 (30–45)	32 (29–45)	36 (30–45)	0.428
Age group				0.576
< 40 years	184 (60.9)	14 (66.7)	184 (60.9)	
≥ 40 years	118 (39.1)	7 (22.3)	118 (39.1)	
Body mass index, kg/m^2^	22.6 (20.9–24.7)	25.5 (23.8–28.1)	22.4 (20.8–24.5)	< 0.001
Weight status				< 0.001
< 24 kg/m^2^	195 (64.6)	5 (23.8)	195 (64.6)	
≥ 24 kg/m^2^	107 (35.4)	16 (76.2)	107 (35.4)	
Profession				< 0.001
Nurse	118 (39.0)	0 (0.0)	118 (42.0)	
Medical technician	47 (15.6)	3 (14.3)	44 (15.7)	
Administrative staff and others^2^	137 (45.4)	18 (85.7)	119 (42.3)	
Shift status				0.005
Non-shift worker	203 (67.2)	20 (95.2)	183 (65.1)	
Shift worker	99 (32.8)	1 (4.8)	98 (34.9)	

Data are summarized as n (%) or median (interquartile range, as appropriate. ^1^For between-group comparisons, the independent samples *t*-test or Mann–Whitney *U* test is used as appropriate. ^2^The “others” included mainly staff at payment counters and research assistants and a small number of those who were neither nurses nor medical technicians.

Females had significantly lower median BMI, proportion of overweight, proportion of administrative staff and others, and non-shift working compared with males.

### Changes in outcome parameters for the whole cohort

Notably, we observed statistically significant differences in all parameters between before and after the intervention (Table 2). Specifically, among the anthropometrics, the program led to significant decreases in BMI, WC, WHR, and WHtR (all *p* < 0.05). Likewise, we observed decreased BF% and VFA and increased MW (*p* < 0.05). Regarding PF, we observed significantly better performance at post-intervention in four parameters than at pre-intervention (all *p* < 0.05), except for sit-and-reach (*p* = 0.359).

**TABLE 2:** Changes in outcome parameters for whole cohort (*n* = 302).

Variable	Pre-intervention	Post-intervention	Change^1^	*p*-value^2^
**Anthropometrics**				
Body mass index (kg/m^2^)	22.6 (20.9–24.7)	22.4 (20.6–24.8)	−0.19 (−0.59–0.18)	< 0.001
Waist circumference (cm)	75 (69–81)	74 (68–81)	−1 (−4–3)	0.014
Waist-hip ratio	80.2 (75.9–84.9)	78.9 (74.7–83.8)	−1.0 (−4.0–1.9)	< 0.001
Waist-to-height ratio	47.1 (43.4–51.0)	46.5 (43.0–50.4)	−0.8 (−2.6–1.6)	0.001
**Body composition**				
Body fat percentage (%)	29.8 (4.7)	29.1 (4.8)	−0.65 (1.19)	< 0.001
Muscle weight (kg)	36.5 (34.0–39.4)	36.7 (31.2–39.9)	0.2 (−0.3–0.6)	< 0.001
Visceral fat area (cm^2^)	60.5 (41.8–79.3)	56.0 (38.8–78.0)	−3.0 (−7.0–1.0)	< 0.001
**Physical fitness**				
Grip strength (kg)	27.0 (23.2–31.0)	27.0 (24.0–31.0)	0.0 (−2.0–2.0)	0.035
High jump (cm)	32.0 (28.0–36.5)	33.0 (29.0–38.0)	2.0 (−1.5–4.0)	< 0.001
Sit-up (count)	26 (20–33)	29 (23–35)	2 (0–4)	< 0.001
Sit-and-reach (cm)	32.1 (26.7–38.5)	32.3 (27.0–38.0)	0.0 (−1.9–2.2)	0.359
Step test	58.4 (53.9–64.3)	60.8 (56.3–66.7)	2.2 (−2.6–6.4)	< 0.001

Data are summarized as mean (standard deviation) or median (interquartile range), as appropriate. ^1^Post-intervention minus pre-intervention. ^2^Paired sample *t*-test for normally distributed continuous variables or Wilcoxon signed rank test.

Furthermore, females had significant improvements in anthropometrics, body composition, and three items of physical fitness (high jump, sit-up, step test) after intervention (Supplementary Table 1) (*p* < 0.05).

### Changes in outcome parameters stratified by sex and age

**FIGURE 3:**
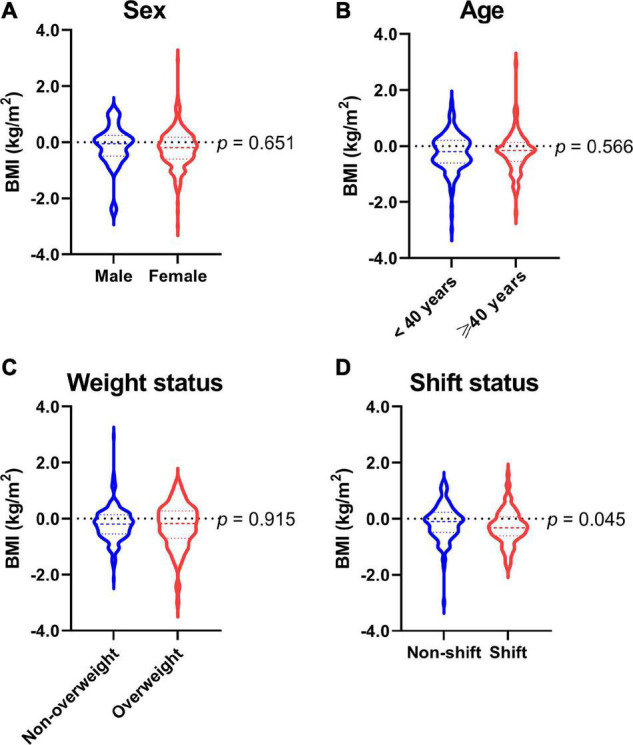
Response differences in body mass index of **(A)** sex subgroups, **(B)** age subgroups, **(C)** weight status subgroups, and **(D)** shift status subgroups. BMI, body mass index. Data are summarized as medians, quartiles, and ranges (violin plots) and compared using generalized estimating equation analyses adjusted for age, profession, and shiftwork status.

Changes from pre-intervention to post-intervention stratified by sex and age were analyzed with the GEEs (Table 3). After adjustment of the other general characteristics, there was no sex difference in the improvements of anthropometrics (BMI demonstrated in Figure 3A), BC, or PF parameters. No significant differences were observed either between age subgroups (BMI demonstrated in Figure 3B).

**TABLE 3:** Changes in outcome parameters stratified by sex and age.

Variable	Male	Female	*p*-value^1^	Age < 40 years	Age ≥ 40 years	*p*-value^1^
**Anthropometrics**						
Body mass index (kg/m^2^)	−0.1 (−0.5–0.2)	−0.2 (−0.6–0.2)	0.651	−0.2 (−0.6–0.2)	−0.2 (−0.6–0.1)	0.566
Waist circumference (cm)	1 (−4–4)	−1 (−4–3)	0.733	−1 (−4–2.9)	−2 (−4–3)	0.829
Waist-hip ratio	0.6 (−2.4–1.9)	−1.0 (−4.1–2.0)	0.548	−1.0 (−4.1–1.7)	−0.9 (−3.7–2.9)	0.777
Waist-to-height ratio	0.6 (−2.4–1.9)	−0.9 (−2.6–1.5)	0.675	−0.5 (−2.5–1.4)	−1.2 (−2.7–1.7)	0.739
**Body composition**						
Body fat percentage (%)	−0.3 (−1.6–0.4)	−0.6 (−1.3–0.1)	0.969	−0.6 (−1.3–0.2)	−0.6 (−1.3–0.1)	0.510
Muscle weight (kg)	0.5 (0.1–1.3)	0.2 (−0.3–0.6)	0.105	0.2 (−0.3–0.6)	0.3 (−0.2–0.7)	0.158
Visceral fat area (came)	−1.0 (−8.0–4.0)	−3.0 (−7.0–0.0)	0.157	−2.0 (−0.2–0.7)	−4.0 (−8.0–1.0)	0.281
**Physical fitness**						
Grip strength (kg)	1.0 (−1.5–4.0)	−2.0 (−3.0–2.0)	0.642	0.0 (−1.0–2.0)	0.0 (−2.0–2.0)	0.820
High jump (cm)	0.0 (−3.0–8.0)	2.0 (−1.0–4.0)	0.721	2.0 (−1.0–4.0)	1.0 (−2.0–4.0)	0.776
Sit-up (count)	3 (−1–5)	2 (0–4)	0.484	2 (0–4)	2 (0–5)	0.858
Sit-and-reach (cm)	−0.8 (−1.2–5.0)	0.1 (−1.9–2.2)	0.345	0.0 (−1.7–2.1)	0.0 (−2.2–3.0)	0.746
Step test	3.7 (−1.4–7.5)	2.0 (−2.6–6.3)	0.332	2.2 (−2.1–6.2)	2.0 (−3.1–6.6)	0.906

Data are summarized as median change (interquartile range). ^1^The difference in change (post-intervention minus pre-intervention) between weight or shiftwork subgroups obtained from generalized estimating equation analyses adjusted for sex, age, profession, and/or shiftwork status. The difference in change (post-intervention minus pre-intervention) between sex or age subgroups obtained from generalized estimating equation analyses adjusted for age, BMI at pre-intervention, profession, and shiftwork status.

Among females, there was no age difference in the improvements of variables of interest after adjustment of the other general characteristics (Supplementary Table 2).

### Changes in outcome parameters stratified by weight and shiftwork status

Changes in outcome parameters stratified by weight status and shiftwork status were analyzed with the GEEs (Table 4). We observed no significant difference in the changes of parameters between the non-overweight and overweight subgroups (BMI demonstrated in Figure 3C). Albeit non-significant, we did observe a borderline (*p* < 0.10) such that participants with overweight showed a slightly greater improvement in step test performance compared to those with non-overweight (3.0 vs. 1.6).

**TABLE 4:** Changes in outcome parameters stratified by weight status and shiftwork status.

Variable	Non-overweight	Overweight	*p*-value^1^	Non-shift worker	Shift worker	*p*-value^1^
**Anthropometrics**						
Body mass index (kg/m^2^)	−0.2 (−0.6–0.1)	−0.2 (−0.7–0.3)	0.915	−0.1 (−0.6–0.2)	−0.3 (−0.6–0.1)	0.045
Waist circumference (cm)	−1 (−4–3)	−1 (−4–3)	0.672	−1 (−4–3)	−1 (−3–3)	0.658
Waist-hip ratio	−1.0 (−4.0–2.1)	−0.8 (−4.0 –1.7)	0.855	−1.3 (−4.7–2.1)	−0.8 (−3.2–1.7)	0.340
Waist-to-height ratio	−0.9 (−2.6–1.6)	−0.8 (−2.4–1.4)	0.691	−0.9 (−2.6–1.6)	−0.7 (−2.5–1.5)	0.626
**Body composition**						
Body fat percentage (%)	−0.7 (−1.4–0.1)	−0.6 (−1.1–0.2)	0.239	−0.7 (−1.3–0.1)	−0.5 (−1.4–0.1)	0.290
Muscle weight (kg)	0.2 (−0.3–0.6)	0.2 (−0.3–0.7)	0.858	0.2 (−0.3–0.7)	0.2 (−0.4–0.5)	0.299
Visceral fat area (came)	−2.0 (−6.0–0.0)	−4.0 (−0.8–2.0)	0.197	−3.0 (−7.0–0.0)	−2.0 (−7.0–1.0)	0.571
**Physical fitness**						
Grip strength (kg)	0.0 (−1.0–2.0)	1.0 (−2.0–3.0)	0.594	0.0 (−1.3–2.0)	0.0 (−2.0–2.0)	0.902
High jump (cm)	1.0 (−1.0–4.0)	2.0 (−2.0–5.0)	0.608	2.0 (−2.0–5.0)	1.7 (−1.0–4.0)	0.231
Sit-up (count)	2 (0–4)	2 (0–5)	0.471	2 (0–4)	3 (−1–5)	0.367
Sit-and-reach (cm)	0.1 (−2.1–2.0)	0.0 (−1.5–2.8)	0.235	−0.1 (−1.9–2.2)	0.2 (−1.8–2.2)	0.999
Step test	1.6 (−2.8–6.5)	3.0 (−1.7–6.1)	0.067	2.1 (−2.2–6.9)	2.5 (−3.0–5.5)	0.880

Data are summarized as median change (interquartile range). ^1^The difference in change (post-intervention minus pre-intervention) between weight or shiftwork subgroups obtained from generalized estimating equation analyses adjusted for sex, age, profession, and/or shiftwork status.

There was a significant difference in the improvement of one parameter—BMI—between participants who worked in shifts and who did not (*p* < 0.05), after adjustment for gender, age, BMI at pre-intervention, and profession. Specifically, while the BMI for the shift and non-shift groups both decreased from pre-intervention to post-intervention, the improvement was substantially greater in the shift group than in the non-shift group (−0.35 vs. −0.19 kg/m^2^) (Figure 3D). No significant difference was observed in the changes of other parameters across shift status. Furthermore, these characteristic differences in the improvements of variables of interest between shift workers and non-shift workers persisted after adjustment of the other general characteristics among females (Supplementary Table 2).

## Discussion

The overall outcome suggested that the program was effective for improving various anthropometric, BC, and PF parameters. Specifically, we observed significant reductions in BMI, WC, WHR, WHtR, BF%, and VFA, along with an increase in MW, indicating a more preferable BC and fat distribution. As for PF, we noted significant improvements in the explosive muscle strength of the upper and lower limbs, muscular endurance, flexibility, and cardiorespiratory endurance. All of these results are compatible with those of prior studies on health promotion exercises (52–56).

Among all the parameters, we observed no significant differences in intervention effects by sex or age, suggesting that these characteristics have no impact on the intervention outcome. This finding is congruent with those of other studies (57, 58). However, while it did not reach the level of statistical significance, we did observe an interesting tendency for sex: the female sex appeared to show a greater improvement, especially in weight status and BC. Other studies have shown that women had better responses than did men after an exercise intervention (59, 60). One study in 2015 showed similar results to ours; wherein women showed greater improvements in anthropometrics than did men (61). However, another study in 2007 suggested that men showed greater improvements than did women in terms of anthropometrics and BC. Considering these previous inconsistent findings and the lack of a meta-analysis, it remains unclear whether health promotion exercise programs directed at medical personnel show sex differences in their effects. Moreover, our study cohort comprised mainly women (93.0%). According to national data, the number of female HCWs has been persistently much higher than that of males. This data truly reflected the fact that the male/female ratio ranged between 6.0% and 25.2% (mean = 14.4%) in our WHP program from 2014 to 2021 as a result of a higher proportion of women working in the healthcare industry and their higher engagement in WHPs. Future studies that focus specifically on sex differences in intervention response may help design sex-specific interventions.

In the present study, we categorized our cohort into “age < 40 years” and “age ≥ 40 years.” According to a large prospective cohort study published in 2019, maintaining higher levels of leisure-time physical activity in adults ≥ 40 years old was associated with a comparative low risk for all-cause mortality as it in adults < 40 years old (62). Saint-Maurice et al. have recommended that the age of 40 years is a considerable important timepoint to start physical activity (62). We’ve further analyzed the differences in variables of interest in three age groups: “18–29 years,” “30–39 years,” and “> 40 years” and found no significant differences existed (data not shown). Therefore, our results supported that the multidisciplinary WHP program can provide comparable benefits of anthropometrics, body composition, and physical fitness to participants with ages ≥ 40 years and < 40 years.

Weight status also did not appear to influence the intervention effects. Notably, the subjects with a higher BMI improved to a larger degree than did those with a lower BMI in cardiorespiratory endurance, with a difference almost reaching statistical significance. This somewhat coincides with previous studies, which have indicated that subjects with higher BMIs tend to respond better to exercise interventions in terms of anthropometrics, BC, and cardiorespiratory function (63, 64). Although it is unclear why the response differences existed, possible reasons include individuals with higher BMI having enhanced motivation and compliance with the program, or perhaps an altered physiological response to the intervention co-existing with the obesity itself. Further investigation would be of interest in future studies.

There was a significant difference in BMI improvement between the shiftwork status subgroups. Specifically, the group who engaged in shiftwork showed a more profound weight reduction than did the other subgroup. The association between shiftwork and higher BMI has been robustly demonstrated in the past (65–68). Sleep duration/quality, timing of meals, and timing of light exposure are usually disrupted for shift workers, thereby leading to disruption of circadian rhythms; such a disruption, in turn, can lead to an increased risk of impaired cardio-metabolic function and associated diseases including obesity, diabetes, and cardiovascular disease (69). The underlying pathophysiology of this effect has been suggested to be multifactorial, including inappropriate meal timing (e.g., breakfast skipping or late meals, which can lead to the lower thermic effect of feeding), altered food preferences (e.g., more sugar-sweetened food, which can lead to a higher total calorie intake) (70), and decreased energy expenditure (69). The results of our study imply that while shift workers tend to have higher BMIs, they appear to be able to lower that BMI more efficiently through interventions. We found this effect to be logical, given that the intervention targeted a healthier diet and regular exercise, which can correct the altered eating behavior and decreased energy expenditure caused by the circadian disruption of shift workers. This may be an important finding and suggests that a multidisciplinary WHP program targeting nutrition and exercise is particularly effective and should be prioritized for shift workers.

Overall, this workplace-based intervention appeared to be effective—not only for weight-status outcomes but also for the improvements of BC, fat distribution, and PF. We suggested that the program being multi-faceted, *trans-*disciplinary, and easy-to-access might have contributed to its success. The literature has suggested that the phases of WHP include: needs assessment, planning, implementation, and evaluation (71). Lack of participation is one of the biggest challenges of WHP, especially in the healthcare industry (72). The authors included participants only above a certain level of participation since the current study aimed to assess the effectiveness and response differences to the multidisciplinary intervention. Including all participants with all-ranged participation levels would mix the issues of participation and intervention effectiveness thus possess a threat to internal validity of the research. Incentives and barriers to participation or methods to improve them were not the focus of this study; however, they are certainly critical to the overall success of a WHP and warrant further research. Moreover, mental health is also vital to the well-being of HCWs, and the inclusion of psychological health promotion such as mindfulness-based programs may further broaden the scope of WHP (73).

The advantage of this study includes its comprehensive assessment of anthropometrics and PF. However, there are some limitations. First, since participants volunteered to participate, rather than being selected randomly or with a stratified method from the correspondent population, the generalizability of this program may be limited. Second, the program was conducted in northern Taiwan and might not be representative of the rest of Taiwan. Third, the intervention did not include psychological programs, which should also be considered in future WHPs. Fourth, the study was an efficacy-based research, which is limited in delineating the effects of intervention designs on outcomes. Future prospective research can be more evaluation-based, so lived experiences, feasibility and acceptability of the intervention, and mechanisms of impact in context can be examined. A larger sample size with a control group and a balanced sex distribution can also contribute to a better understanding on the effectiveness of and response differences in WHP among HCWs.

In conclusion, the workplace-based health promotion exercise program was an effective intervention model for hospitals regardless of unfavorable workplace settings for health promotion. Workers with higher BMIs, who engage in shiftwork, and who are at greater risk yet appear more responsive to interventions than their peers, can be considered a priority group for WHP in case of limited resources. Future investigation of response differences and their underlying mechanisms across sex, age, weight, and shiftwork statuses would be interesting.

## Data availability statement

The original contributions presented in the study are included in the article/Supplementary material, further inquiries can be directed to the corresponding author/s.

## Ethics statement

The studies involving human participants were reviewed and approved by Institutional Review Board of Chang Gung Medical Foundation. The patients/participants provided their written informed consent to participate in this study.

## Author contributions

JJ-CL and H-HC conceived and planned the study. C-TC and JJ-CL enrolled the patients. L-AL and H-HC designed the study, analyzed data, made the statistics, and interpreted results. K-HC, N-KW, C-TC, L-AL, and H-HC participated in manuscript drafting. C-YH, Y-AL, and JJ-CL supervised the study. All authors read and approved the final manuscript.
