# Supplementary material for: Effectiveness and response differences of a multidisciplinary workplace health promotion program for healthcare workers
Source: Front Med (Lausanne). 2022 Jul 26;9:930165. doi: 10.3389/fmed.2022.930165 (PMC9360800; doi:10.3389/fmed.2022.930165)
Supplement: Supplementary file 1 [file Data_Sheet_1.pdf]

Supplementary Tables

Supplementary Table 1 | Changes in outcome parameters for whole cohort (*n* = 281).

Variable	Pre-intervention	Post-intervention	Change[1]	*p*-Value[2]
**Anthropometrics**				
Body mass index (kg/m^2)	22.4 (20.8–24.5)	22.2 (20.5–24.3)	-0.19 (-0.60–0.17)	< 0.001
Waist circumference (cm)	74 (69–80)	74 (68–79)	-1 (-4–3)	0.011
Waist-hip ratio	79.5 (75.3–84.2)	78.5 (74.4–83.4)	-1.0 (-4.0–2.0)	< 0.001
Waist-to-height ratio	46.8 (43.3–50.5)	46.2 (42.7–50.0)	-0.9 (-2.6–1.5)	0.001
**Body composition**				
Body fat percentage (%)	30.0 (4.6)	29.4 (4.7)	-0.65 (1.15)	< 0.001
Muscle weight (kg)	36.1 (33.9–38.7)	36.3 (33.9–38.7)	0.2 (-0.3–0.6)	< 0.001
Visceral fat area (cm^2)	57.0 (41.0–77.0)	3.0 (37.0–75.0)	-3.0 (-7.0–0.0)	< 0.001
**Physical fitness**				
Grip strength (kg)	27.0 (23.0–30.0)	27.0 (24.0–30.5)	0.0 (-2.0–2.0)	0.074
High jump (cm)	32.0 (27.0–36.0)	33.0 (29.0–37.0)	2.0 (-1.0–4.0)	< 0.001
Sit-up (count)	26 (20–32)	28 (22–34)	2 (0–4)	< 0.001
Sit-and-reach (cm)	32.8 (27.6–38.8)	32.8 (27.9–38.1)	0.1 (-1.9–2.2)	0.473
Step test	58.4 (53.9–64.7)	60.8 (56.3–67.2)	2.0 (-2.6–6.3)	< 0.001

Data are summarized as mean (standard deviation) or median (interquartile range), as appropriate. [1]Post-intervention minus pre-intervention; [2]Paired sample t-test for normally distributed continuous variables or Wilcoxon signed rank test.

Supplementary Table 2 | Changes in outcome parameters stratified by age, weight status, and shiftwork status.

Variable	Age < 40 years	Age ≥ 40 years	*p*-Value[1]	Non-overweight	Overweight	*p*-Value[1]	Non-shift worker	Shift worker	*p*-Value[1]
**Anthropometrics**									
Body mass index (kg/m^2)	-0.2 (-0.6–-0.2)	-0.2 (-0.6–-0.2)	0.357	-0.2 (-0.5–0.1)	-0.2 (-0.8–0.3)	0.877	-0.1 (-0.6–0.2)	-0.3 (-0.6–0.0)	0.040
Waist circumference (cm)	-1 (-4–2)	-1 (-4–3)	0.279	-1 (-4–3)	-1 (-4–2)	0.651	-1 (-4–3)	-1 (-4–3)	0.704
Waist-hip ratio	-1.2 (-4.3–1.7)	-0.8 (-3.3–3.2)	0.123	-1.0 (-4.1–2.1)	-1.0 (-4.0–1.7)	0.801	-1.5 (-4.7–2.1)	-0.8 (-3.3–1.7)	0.287
Waist-to-height ratio	-0.8 (-2.6–1.2)	-0.1.0 (-2.6–1.8)	0.321	-0.8 (-2.6–1.7)	-1.0 (-2.6–1.3)	0.639	-0.9 (-2.6–1.5)	-0.7 (-2.5–1.4)	0.643
**Body composition**									
Body fat percentage (%)	-0.7 (-1.3–0.1)	-0.6 (-1.2–0.1)	0.427	-0.7 (-1.4–0.1)	-0.6 (-1.1–0.1)	0.272	-0.7 (-1.3–0.1)	-0.5 (-1.3–1.3)	0.922
Muscle weight (kg)	0.1 (-0.4–0.6)	0.3 (-0.2–0.7)	0.168	0.2 (-0.3–0.6)	0.1 (-0.4–0.7)	0.914	0.2 (-0.3–0.7)	0.2 (-0.4–0.5)	0.277
Visceral fat area (came)	-2.0 (-6.0–0.0)	-4.0 (-8.0–1.0)	0.294	-2.0 (-6.0–0.0)	-4.0 (-8.0–1.0)	0.357	-3.0 (-7.0–0.0)	-2.0 (-7.0–1.0)	0.350
**Physical fitness**									
Grip strength (kg)	0.0 (-1.3–2.0)	0.0 (-2.0–2.0)	0.868	0.0 (-1.3–2.0)	0.0 (-2.0–2.0)	0.556	0.0 (-2.0–2.0)	0.0 (-2.0–2.0)	0.579
High jump (cm)	2.0 (-1.0–4.0)	2.0 (-1.0–4.0)	0.779	1.0 (-1.0–4.0)	2.0 (-1.0–4.0)	0.973	2.0 (-1.0–4.0)	1.2 (-1.0–4.0)	0.222
Sit-up (count)	2 (0–4)	2 (0–4)	0.978	2 (0–4)	2 (0–4)	0.724	2 (0–4)	3 (0–5)	0.666
Sit-and-reach (cm)	0.1 (-1.8–2.0)	0.0 (-2.1–3.0)	0.592	0.1 (-2.1–1.9)	0.2 (-1.7–2.3)	0.371	-0.1 (-2.1–2.2)	0.3 (-1.8–2.3)	0.856
Step test	2.2 (-2.2–6.3)	1.5 (-.31–6.6)	0.745	1.6 (-2.8–6.4)	2.9 (-1.9–6.1)	0.498	2.0 (-2.6–6.8)	2.5 (-2.8–5.6)	0.449

Data are summarized as change (95% confidence interval). [1]The difference in change (post-intervention minus pre-intervention) between age, weight or shiftwork subgroups obtained from generalized estimating equation analyses adjusted for age, BMI at pre-intervention, profession, and shiftwork status.

Supplementary Figures

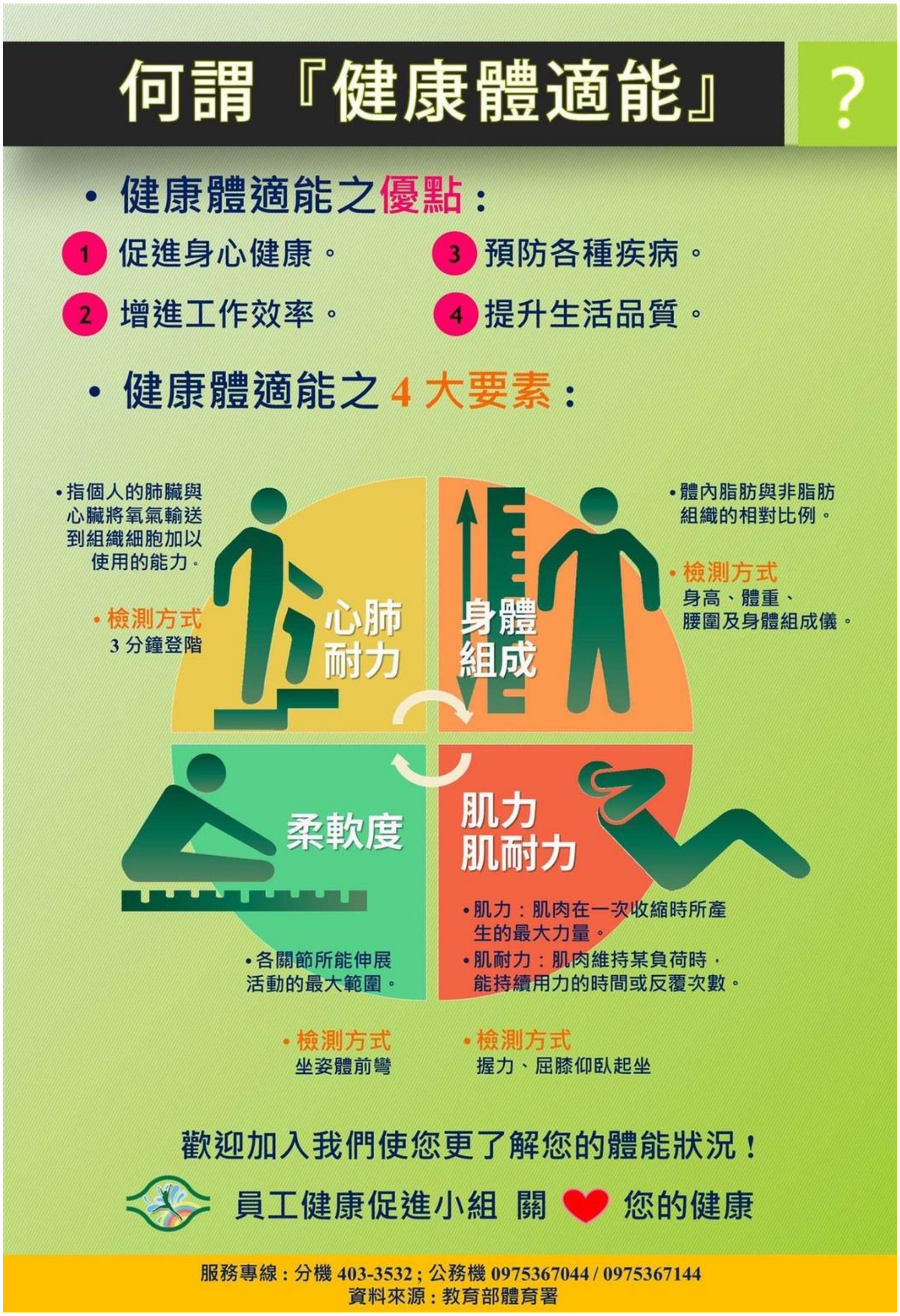

Supplementary Figure 1 | Educational material about healthy PF.

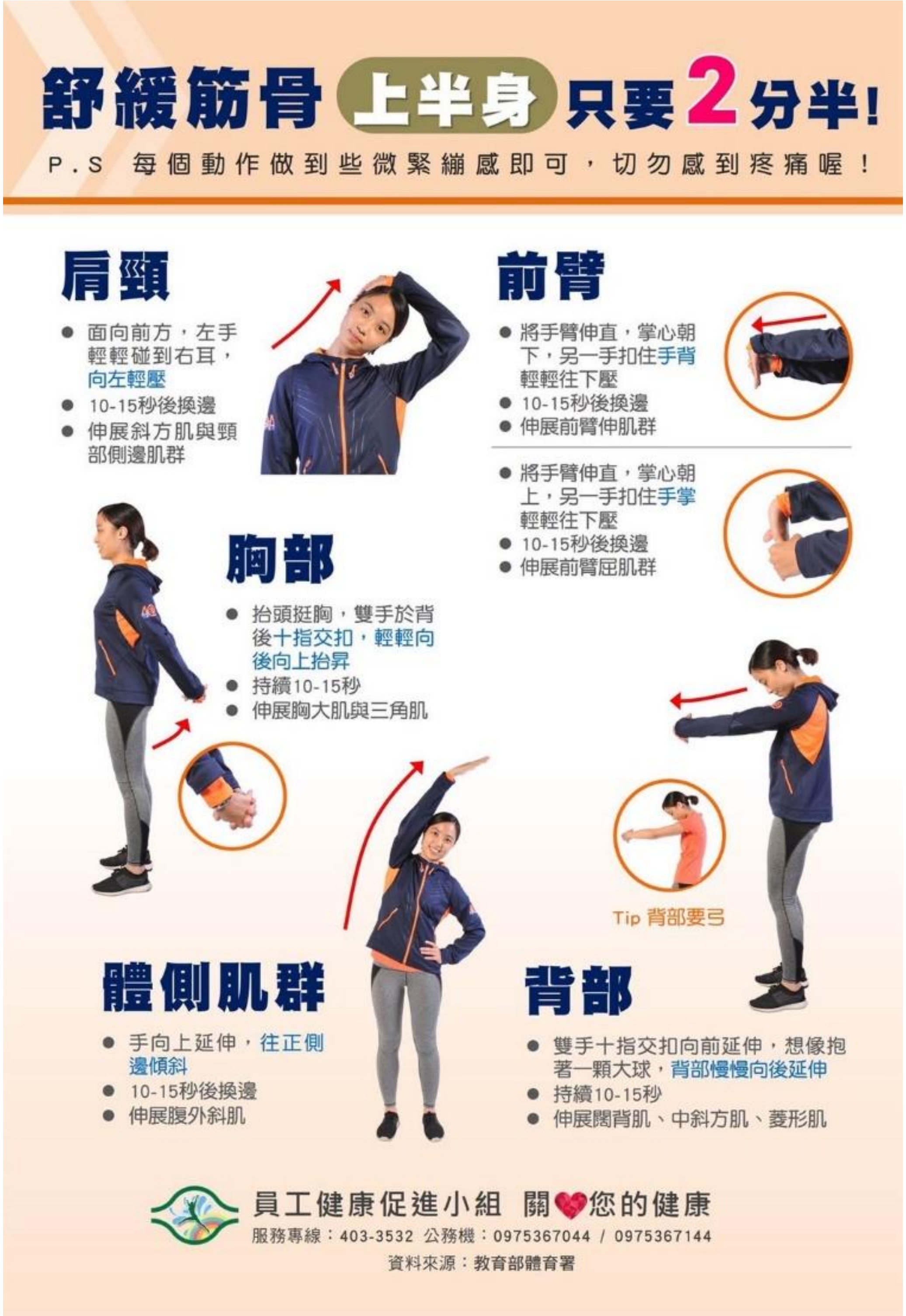

Supplementary Figure 2 | Educational material about stretching exercise.

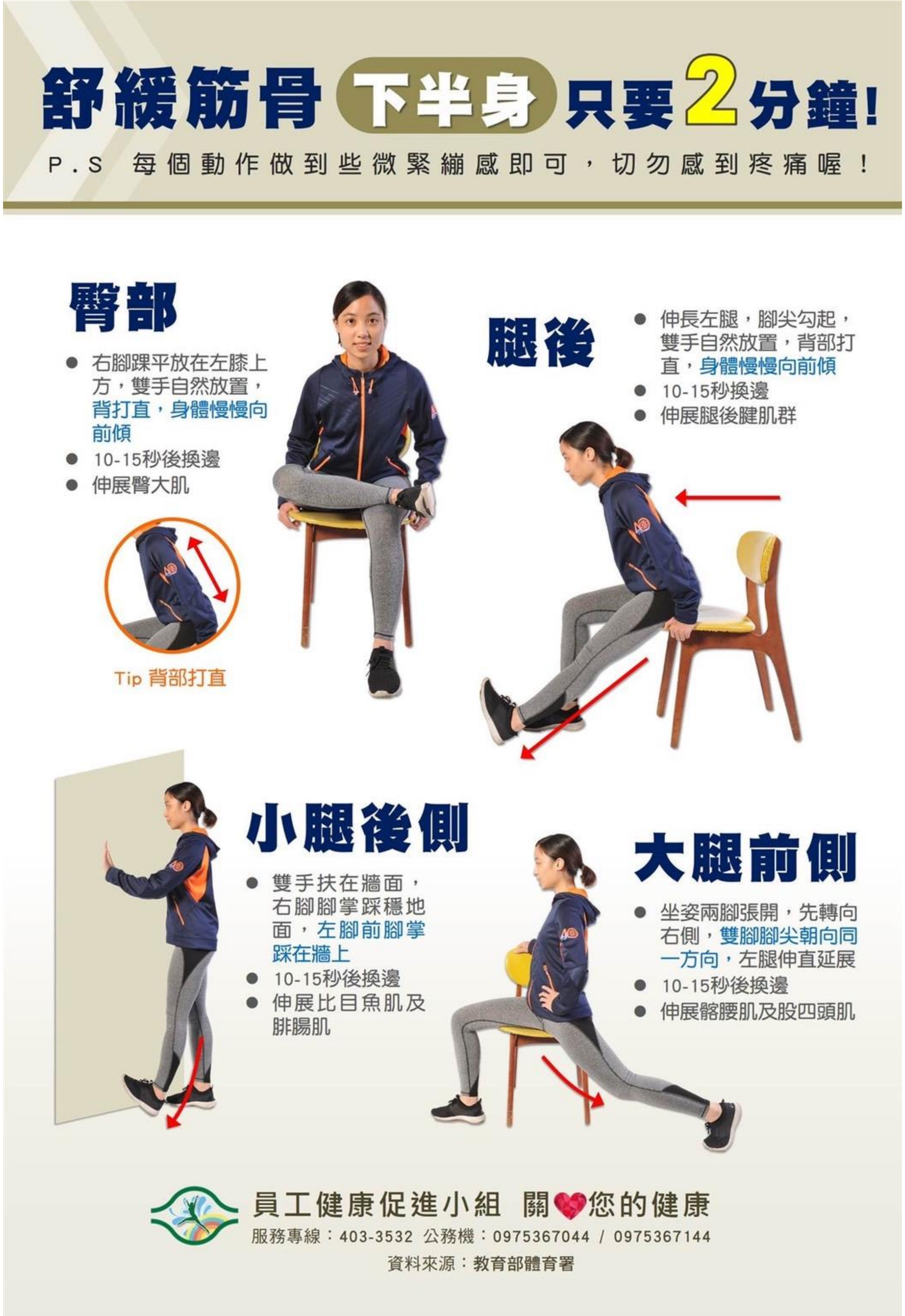

Supplementary Figure 3 | Educational material about stretching exercise.

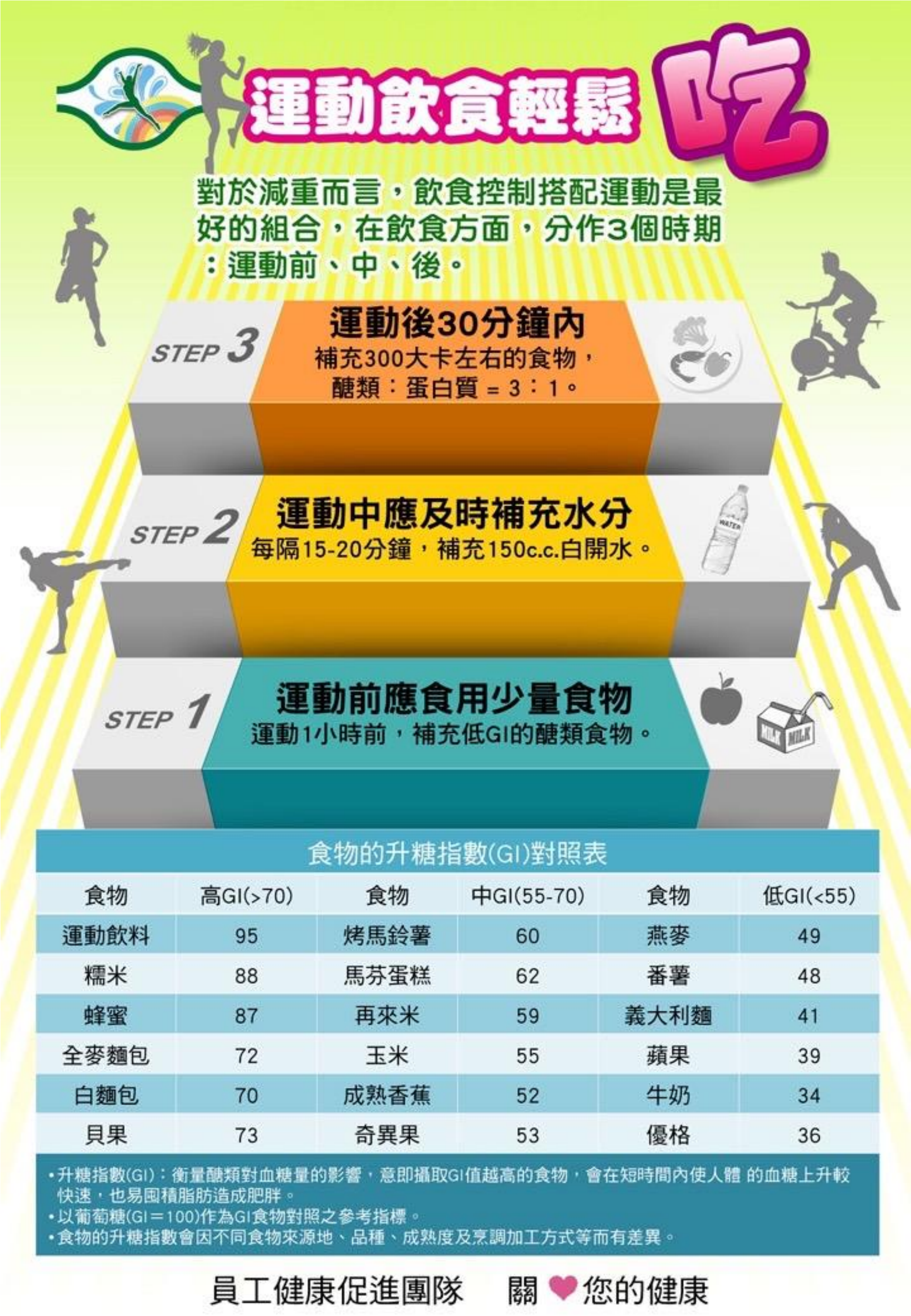

Supplementary Figure 4 | Educational material about diet and exercise for weight management.
